# A Case Presenting Many Interesting Features*Read at November meeting, Brazos Valley Medical Association.

**Published:** 1907-01

**Authors:** R. E. B. Bledsoe

**Affiliations:** Somerville, Texas


For Texas Medical Journal.

### A Case Presenting Many Interesting Features.*

DR. R. E. B. BLEDSOE, SOMERVILLE, TEXAS.

July 23, 1906, I was called to see H. Sheffield, an express messenger, age about 21 years, for chills and fever. He gave a history of a chill every day about 12 o'clock or a little earlier each day, for the past three days and upon a microscopic examination I found the blood quite rich in malarial organisms of the tertian type, and as paroxysms occurred daily it was evident he had a double tertian infection, or, as the Germans say, a malignant tertian fever.

After three days of 60 grains doses of quinine per diem, the

*Read at November meeting, Brazos Valley Medical Association.

chills and high fever of malaria subsided, temperature reaching 99 degrees F. on the morning of the fourth day, when I dismissed the case. Two days later I was called to same patient for fever which reached its highest point in the afternoon, subsiding in the night, and leaving him free of fever in the mornings, as he expressed it.

On the morning of the seventh day, after my first visit, patient's temperature was 99.2° F., and in the afternoon, about 6, temperature reached 102.3° F. He had had several stools that day and upon inspection the stool presented what I am in the habit of calling a characteristic typhoid appearance; this, in connection with the appearance of the tongue and the behavior of the temperature, warranted me in making a diagnosis of typhoid to which the patient strenuously objected.

The family with whom this young man boarded informed me that he refused to use the bed pan, insisted on something to eat all the time and, in fact, was such an unruly fellow that they couldn't hold him down. We endeavored to get a trained nurse at this juncture, but failed. I talked to him, explained the necessity of quietude, the folly of getting out of bed to use vessel, but to no purpose, the family said, for as soon as I would leave he would insist on something to eat and that I didn't know what I was talking about, that he knew he didn't have any typhoid fever.

About this time he had a hemorrhage and I withdrew everything by way of mouth, put ice bags on abdomen and administered opium.

I was enabled to keep the patient reasonably quiet and still for two days and nights after the hemorrhage and have him pass his urine without springing up, although he said he had had no hemorrhage and that he had no typhoid fever.

Some three or four days after the hemorrhage the fever left him, tongue cleared off, he became ravenously hungry, slept well and felt fine, as he said. I saw him for a day or two longer and again dismissed the case, after explaining the absolute necessity of quietude and the avoidance of all solid foods.

I have said nothing of the treatment in this case because typhoid patients need no medicine; they do as well, and I believe better, without medicine, that is, as a routine matter.

In my opinion temperature and diet are the only things to be considered in typhoid, uncomplicated by hemorrhage.

Some five or six days after the second dismissal I was called again to see patient, found him with temperature 101, tongue foul, bowels tympanitic and loose, some characteristic stools. I notified his father at Alvin of the relapse and he came, bringing a trained nurse. Case went along nicely for several days, nurse gaining absolute control over patient. The sixth or seventh day after the relapse, patient had a small hemorrhage, which was controlled or rather treated in the manner indicated above. Patient now complained of great pain and tenderness about McBurney point; however, the other symptoms about same, with no further hemorrhage. Pain and tenderness about McBurney point persisting, caused me to consider the possibility of appendicitis very carefully.

On the morning of September 3, 1906, about 6:30, I was called by nurse to see patient with a chill which had been immediately preceded and was accompanied by a sharp stabbing pain in right flank.

I made a diagnosis of a typhoid perforation and explained to the young man that it would be necessary to go into his abdomen at once to which he readily consented. My partner, Dr. Moses, saw the case with me when we carried up our operating paraphernalia and agreed that an operation was imperative. I told Dr. Moses that we would likely find mischief of some sort in appendix, but that I felt that we had to deal with a typhoid perforation. By 1 o'clock we were into abdominal cavity by the McBurney route and when we opened the peritoneum, the first thing that presented was the ilium with a typhoid perforation sufficiently large to admit the blunt end of the scalpel, situated about three inches from caput coli on free margin with a typhoid ulcer about three-fourths of an inch further down ilium, almost at stage of perforation. The perforation was repaired and the ulcer being so close to the perforation was purse-stringed and buttressed to the repair operation. We discussed the advisability of resection and the introduction of a Murphy button before the stitching of wound and also after, but as we were both positive that we had not seriously encroached upon the lumen of the bowel, we did not resect. An interesting feature of the case was a typhoid ulcer in base of appendix which I here present for your inspection. The appendix, as you see, has been preserved in alcohol and is greatly shriveled, but you will have no difficulty in locating the ulcer..

In going up the ilium in search of other possible perforation, I found a Merkel's Diverticulum about the bigness of a thumb, which had participation in the typhoid inflammation.

It was instructive and interesting to note the recession of the typhoid inflammation which was taking place in the ilium higher up. The relapse attack seemed confined to the appendicular region—if you will permit me the expression—as we only found the four ulcers, one in base of appendix, the perforation and its companion, and one in caput coli.

We took patient on table with a temperature of 104+ and a pulse of between 130 and 140; respirations very shallow and rapid.

It requires some little fortitude to operate on a case in the face of such evident symptoms of collapse with a 6 or 7 to 1 shot against you under most favorable surroundings, but I should have always reproached myself had I not operated on this case.

That afternoon at 4 o'clock, patient's temperature was 99° with pulse 100, and for two days and nights he looked to be making a splendid recovery, but on evening of third day after operation, he began sinking, gradually growing weaker, vomited a time or two and died—probably a low grade general peritonitis, as we had mopped out over a quart of liquid bowel contents at time of operation.

